# SWE, C-TIRADS and AI-assisted diagnostic systems in distinguishing thyroid nodules of different lesion size: Which has better diagnostic performance?

**DOI:** 10.3389/fendo.2025.1691092

**Published:** 2026-01-07

**Authors:** Yuwei Zhou, Ruifen Li, Weiwei Zheng, Zhaoguang Liu, Jing Yang, Lei Wang

**Affiliations:** 1 Department of Ultrasound, Tangshan People’s Hospital, Tangshan, China; 2 Department of Pathology, Tangshan People’s Hospital, Tangshan, China

**Keywords:** artificial intelligence-assisted diagnostic system, Chinese thyroid imaging reporting and data system, shear wave elastography, thyroid nodule, ultrasound

## Abstract

**Objective:**

To compare the diagnostic performance of shear wave elastography (SWE), Chinese Thyroid Imaging Reporting and Data System (C-TIRADS), and an artificial intelligence (AI)-assisted diagnostic system in differentiating thyroid nodules of different sizes.

**Methods:**

A total of 103 thyroid nodules in 90 patients were prospectively analyzed and divided into two groups based on the maximum diameter: <10 mm and ≥10 mm. Each thyroid nodule was evaluated using three methods: conventional ultrasound for C-TIRADS scoring, shear wave elastography (SWE), and AI-assisted diagnosis. The diagnostic performance of individual methods and their combinations was assessed within each nodule size group using sensitivity, specificity, and area under the receiver operating characteristic (ROC) curve. For combined assessments, a nodule was considered positive if any constituent method indicated malignancy. Intergroup comparisons of AUC values were performed using DeLong’s test to evaluate the effect of nodule size on diagnostic performance.

**Results:**

In nodules ≥1 cm, AI demonstrated excellent performance (AUC = 0.875, sensitivity = 96.43%, specificity = 77.78%), and C-TIRADS also performed well (AUC = 0.834, sensitivity = 96.55%, specificity = 70.37%). Among SWE parameters, Emax achieved the highest AUC (0.895). The diagnostic efficacy of AI combined with C-TIRADS (AUC = 0.852) was comparable to that of AI + C-TIRADS + Emax. In subcentimeter nodules, diagnostic performance decreased, with AI achieving an AUC of 0.654 and C-TIRADS an AUC of 0.524, whereas Emean retained moderate discriminative ability (AUC = 0.821).

**Conclusion:**

AI combined with C-TIRADS provides an efficient and practical strategy for diagnosing thyroid nodules ≥1 cm. For subcentimeter nodules, Emean retains discriminative ability, indicating potential clinical value in assessment of small lesions.

## Introduction

Thyroid nodules are a common clinical finding, with their global prevalence steadily increasing over the past decades (1–3). According to the Global Cancer Statistics (GLOBOCAN) 2022, thyroid cancer(TC) ascended to the third most prevalent cancer type, with an increase of 245,025 new cases compared to 2020 (1). China accounted for the largest proportion of TC cases worldwide (56.77%) and also reported the highest number of TC-related deaths, representing 24.35% of the global total (4). In the context of this growing epidemiologic burden, accurate and standardized evaluation of thyroid nodules has become a crucial component of clinical management.

Ultrasound is the first-line imaging modality for evaluating thyroid nodules due to its accessibility, non-invasiveness, and diagnostic utility (5–7). In clinical practice, several ultrasound-based risk stratification systems, such as the Chinese Thyroid Imaging Reporting and Data System (C-TIRADS), have been developed to predict malignancy risk and guide the decision for fine-needle aspiration cytology (FNAC) (8–11). These systems rely on a combination of sonographic features, including echogenicity, margin characteristics, shape, and presence of calcifications. However, ultrasound interpretation is inherently operator-dependent, and the diagnostic performance may vary significantly with the physician’s level of experience (12).

FNAC remains the gold standard for thyroid nodule diagnosis, but its accuracy is affected by lesion size and sampling technique (7, 13). Notably, a considerable number of nodules yield indeterminate results (e.g., Bethesda III or TIR3), posing diagnostic challenges and potentially resulting in unnecessary surgeries (14). These limitations have highlighted the need for adjunct diagnostic tools to improve risk stratification and reduce unnecessary interventions.

In recent years, advanced technologies such as shear wave elastography (SWE) and artificial intelligence (AI)-assisted diagnostic systems have emerged as promising tools in thyroid nodule evaluation (15–17). SWE provides a quantitative assessment of tissue stiffness, aiding in the identification of malignancies that typically exhibit increased elasticity (15). AI-assisted systems, based on deep learning algorithms, offer standardized image analysis and have demonstrated high diagnostic consistency, especially among junior physicians (18). Notably, the 2023 edition of the Chinese Guidelines for the Diagnosis and Treatment of Thyroid Nodules and Differentiated Thyroid Cancer (Second Edition) formally incorporated SWE and AI-assisted diagnostic tools into the diagnostic workflow, acknowledging their growing clinical relevance (19).

However, current evidence regarding the impact of nodule size on the diagnostic efficacy of these emerging technologies is heterogeneous and lacks consensus (20, 21). For example, the guidelines of the European Federation of Societies for Ultrasound in Medicine and Biology (EFSUMB) state that SWE is applicable to nodules of all sizes, provided appropriate adjustment of the ROI (22). In contrast, the 2021 guidelines of the World Federation for Ultrasound in Medicine and Biology (WFUMB) suggest that SWE performs more reliably in nodules larger than 10 mm, while its diagnostic value may be compromised in subcentimeter nodules (23). In addition, evidence remains insufficient regarding how SWE, TI-RADS, and AI-assisted systems, particularly across nodules of different sizes. Most previous studies evaluated these modalities independently, and size-specific diagnostic differences have not been systematically characterized (20, 21). These gaps highlight the need to clarify the comparative performance of these tools in subcentimeter and ≥10-mm nodules.

Therefore, the aim of this study was to systematically compare the diagnostic performance of SWE, C-TIRADS, and AI-assisted diagnostic system in assessing thyroid nodules across different size categories. The findings of this study may provide evidence to support the optimal application of these tools in clinical practice and contribute to more personalized and precise management strategies for thyroid nodules.

## Materials and methods

### Study sample

From July 2024 to March 2025,90 patients from the ultrasound department of a tertiary oncology hospital were prospectively collected and analyzed. This study was approved by the hospital’s ethics committee (approval No. RMYY-LLKS-2025309), and informed consent was obtained from all patients. The inclusion criteria were as follows: (1) Age 16–73 years, with no sex preference, (2) All cases presented with thyroid nodules, (3) Definite pathological diagnosis obtained from surgery or fine-needle aspiration (FNA), (4) Underwent conventional ultrasound, SWE, and AI-assisted examinations with clear and complete imaging data, (5) No history of thyroid surgery, biopsy, or thermal ablation therapy. The exclusion criteria were as follows: (1) Lack of pathological confirmation (no surgery or FNA performed, or indeterminate cytology without surgical follow-up), (2) History of other malignant tumors, (3) Poor general condition or inability to cooperate with breath-holding during examination, (4) Incomplete imaging records or clinical data.

### Equipment, instruments, and AI-assisted diagnostic system

A color Doppler ultrasound system (EPIQ 7, Philips, Netherlands) equipped with a linear-array probe (eL18-4) and shear-wave elastography (SWE) software was used for image acquisition. The AI-assisted diagnostic system evaluated in this study, the ITS100 Ultrasound Imaging Intelligent System (Version 1.2; Sichuan Maide Intelligent Technology Co., Ltd., China), consists of a main processing unit and an AI-assisted display module. The system, which was developed using a multicenter dataset of more than 100,000 thyroid ultrasound images from Chinese patients with pathologically confirmed benign and malignant nodules.

### Conventional ultrasound examination

The patient was positioned in the supine position. After identifying the lesion location using two-dimensional grayscale ultrasound, the tumor’s orientation, margin, composition, echogenicity, presence of microcalcifications, anatomical location, position, lymph node metastasis, and maximum diameter were recorded. Additionally, color doppler flow imaging (CDFI) was employed to assess the blood flow grading of the tumor. CDFI utilized the Adler semi-quantitative method, with the following classification (24):Level 0: No blood flow signals detected in the lesion; Level I: One or two “star-like” blood flow signals with a diameter less than 1 mm detected within the tumor; Level II: Three or four spot-like blood flow signals, or a major vessel traversing the tumor; Level III: Four or more blood vessels detected within the lesion.

Image assessments were independently performed by two ultrasound physicians, each with over five years of clinical experience. The interpretation of results was based on the C-TIRADS criteria (10). Each lesion was assigned a score to aid in differentiating benign from malignant lesions (**Table 1**). In cases of discordant opinions, a senior physician with 20 years of experience was consulted, and the final diagnosis was established through consensus, based on the majority opinion. All physicians involved were blinded to the final diagnoses of the thyroid nodules.

**Table 1:** C-TIRADS classification.

C-TIRADS grade	Score	Description	Malignancy risk
C-TIRADS 1	Unscored	Normal	0
C-TIRADS 2	-1	Benign	0
C-TIRADS 3	0	Probably Benign	<2
C-TIRADS 4A	1	Low Suspicion	2-10
C-TIRADS 4B	2	Intermediate Suspicion	10-50
C-TIRADS 4C	3-4	High Suspicion	50-90
C-TIRADS 5	5	Highly Suggestive of Malignancy	>90
C-TIRADS 6	–	Proven Malignancy	100%

### Real-time SWE technology acquisition and analysis

Real-time SWE was conducted on the EPIQ7 ultrasound system via its built-in elastography module. After obtaining the optimal imaging plane of the lesion using gray-scale ultrasound, the mode was switched to ElastQ, and SWE was selected for assessment. The probe was positioned perpendicularly and gently placed on the skin surface, ensuring adequate coupling gel. The sampling box was adjusted to encompass the entire lesion as much as possible, including the target region and surrounding tissues while avoiding the skin and carotid artery. To minimize the impact of respiratory motion on the image, patients were instructed to hold their breath. The best image with optimal color filling was selected for analysis.

The ROI Q-box was placed over the hardest area of the lesion (for heterogeneous lesions, the Q-box was placed within the lesion, avoiding cystic or calcified regions). Another ROI Q-box was placed on the normal thyroid tissue at a depth similar to that of the lesion. The maximum (Emax), mean (Emean), and median (Emed) values of Young’s modulus (in kPa) were recorded, where E represents the elasticity of the tissue (25).Each lesion was measured at least three times by a physician trained in SWE to ensure measurement consistency, and the average value was used.

The optimal cutoff values for SWE parameters (Emax, Emean, and Emed) were determined based on ROC curve analysis of the study dataset. Thresholds were selected using the Youden index and then applied to calculate diagnostic performance metrics, including sensitivity, specificity, PPV, NPV, and accuracy.

### Dynamic AI-assisted diagnostic system

**Figure 1:**
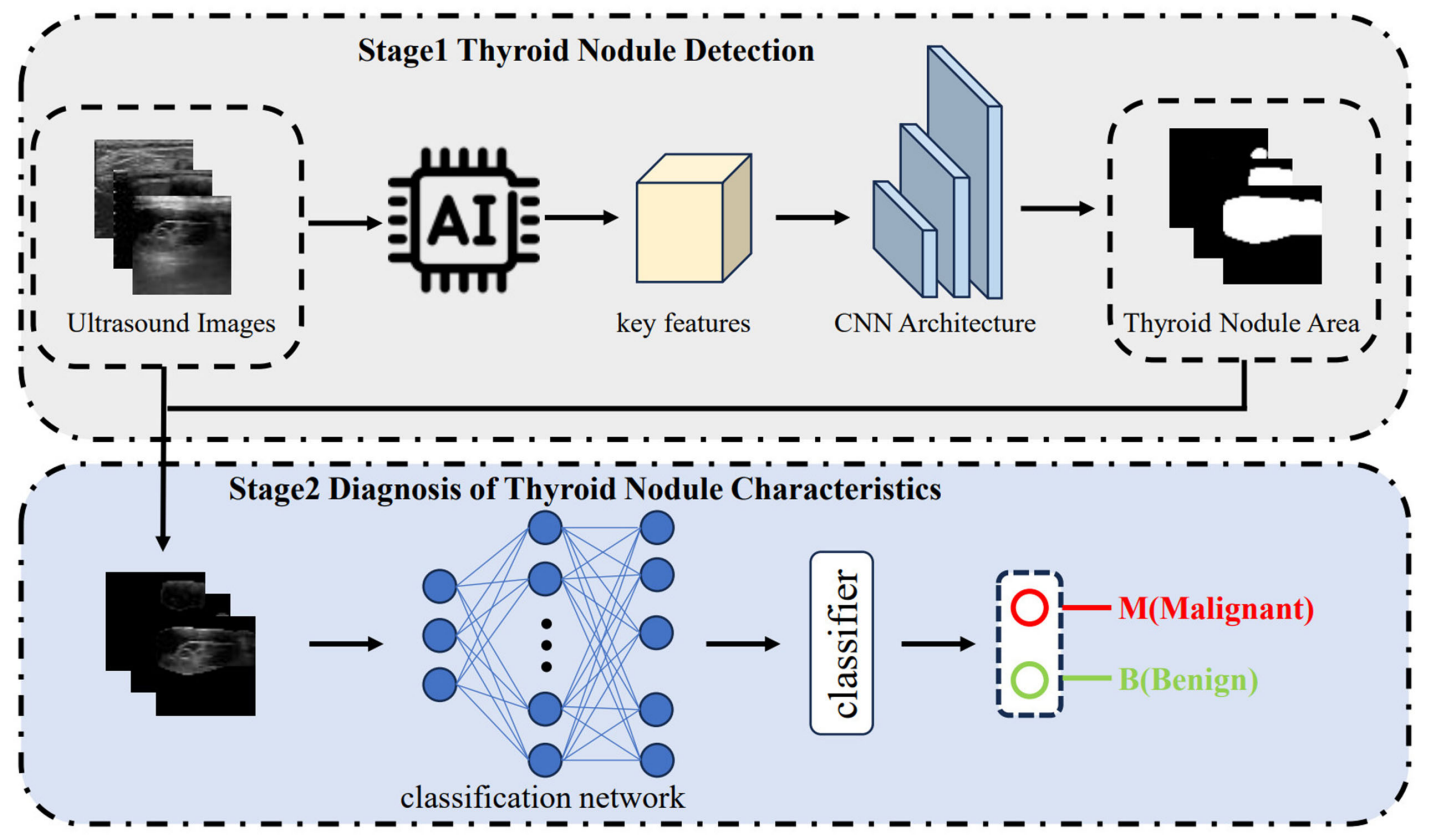
Workflow of the AI-assisted diagnostic system for thyroid nodules. Stage 1: detection of thyroid nodules from ultrasound images using a CNN-based model to automatically localize the nodule area. Stage 2: classification of nodule characteristics through a neural network and classifier to differentiate malignant (M) from benign (B) lesions.

In the event of a detected nodule, the AI leverages deep learning technology to automatically identify key image features. Using the distinctive features of ultrasound images, it crafts a CNN architecture for diagnostic purposes. With the help of CNN’s convolution kernel, the system samples pixels from the input image, extracting global features specific to the thyroid nodule area. This process builds a high-throughput, multi-level characteristic space. Once the diagnostic pattern analyzes and calculates the features of the input nodule’s image, it provides two probability values. These values represent the probability that a thyroid nodule is benign or malignant, as assessed by the diagnostic algorithm. The system outputs a categorical prediction-”malignant” (indicated by a red “M”) or “benign” (indicated by a green “B”)-based on the identified imaging pattern. Nodules classified as “uncertain” (C-TIRADS 4A) by AI are represented with an alternating red and green color pattern. In this study, the single “uncertain” nodule was excluded from the calculation of AI diagnostic performance to avoid verification bias and overestimation of specificity (**Figure 1**).

### Statistical analysis

All statistical analyses were conducted using SPSS software, version 26.0 (IBM Corp., Armonk, NY, USA), with α = 0.05 as the significance level for inter-group comparisons. Continuous data were expressed as mean ± standard deviation ( ¯± s). Categorical and ordinal data were presented as frequencies and percentages (%). For normally distributed continuous data, comparisons between two groups were conducted using independent sample t-tests, whereas the Mann–Whitney U test was used for non-normally distributed data. For categorical data, inter-group comparisons were performed using the chi-square (χ2) test, while the rank-sum test was applied for ordinal variables. Receiver operating characteristic (ROC) curve analysis was performed to assess the diagnostic value of each indicator, determine optimal cut-off points, and evaluate overall diagnostic performance. For ROC analyses involving C-TIRADS, categories were dichotomized as follows: 1–3 = benign, 4A–4C = malignant. This dichotomization was consistently applied for the calculation of sensitivity, specificity, and AUC.

## Results

### Comparison of pathological and clinical data between different nodule size groups

A total of 103 thyroid nodules from 90 patients aged between 16 and 73 years were included in the analysis. All nodules were confirmed by postoperative pathological examination. Based on nodule size, patients were divided into two groups: <10 mm (n = 47) and ≥10 mm (n = 56). The diameters of nodules ranged from 2.9–9.8 mm in the <10 mm group (median = 6.7 mm) and 10.2–46.9 mm in the ≥10 mm group (median = 20.8 mm), with a significant difference between the two groups (Z = –8.714, P < 0.001, Mann–Whitney U test) (**Table 2**). There were no statistically significant differences between the two groups in terms of age (45.87 ± 10.24 vs. 47.86 ± 12.49 years, P = 0.386) or sex distribution (male: 23.41% vs. 21.43%, female: 76.59% vs. 78.57%, P = 0.810) (**Table 2**).

**Table 2:** Comparison of general characteristics.

Variable	Category	<10 mm(n=47)	≥10 mm(n=56)	χ2/t/Z	P
Age(years)		45.87 ± 10.24	47.86 ± 12.49	-0.870	0.386
Sex	Male	11(23.41)	12(21.43)	0.058	0.810
	Female	36(76.59)	44(78.57)		
Nodule Size(mm)		0.67 (0.29–0.98)	2.08 (1.02–4.69)	–8.714	<0.001

Data are presented as mean ± SD, n (%), or median (IQR).

t: independent samples t test; χ²: chi-square test; Z: Mann–Whitney U test.

Among the 47 nodules with a maximum diameter of <10 mm, 41 (87.23%) were diagnosed as papillary thyroid carcinoma, 3 (6.38%) as nodular goiters, 2 (4.25%) as thyroid follicular nodular lesions with focal fibrosis, and 1 (2.12%) as subacute thyroiditis. In contrast, among the 56 nodules with a maximum diameter of ≥10 mm, 28 (50.00%) were papillary thyroid carcinoma, 25 (44.64%) were nodular goiters, 2 (3.57%) were thyroid follicular nodular lesions with focal fibrosis, and 1 (1.78%) was medullary thyroid carcinoma (**Table 3**). In total, 70 nodules were malignant, including 69 papillary thyroid carcinomas and 1 medullary thyroid carcinoma, while the remaining 33 nodules were benign (**Table 3**).

**Table 3:** Patient's pathological result.

Pathological	<10 mm (n=47)	≥10 mm(n=56)
Nodular goiters	3(6.38)	25(44.64)
Thyroid follicular nodular lesions with focal fibrosis	2(4.25)	2(3.57)
Papillary thyroid carcinoma	41(87.23)	28(50.00)
Medullary thyroid carcinoma	0(0)	1(1.78)
Subacute thyroiditis	1(2.12)	0(0)

### Comparison of US parameters between different nodule size groups

The conventional ultrasonographic features of thyroid nodules were compared between the <10 mm (n = 47) and ≥10 mm (n = 56) groups (**Table 4**). Significant differences were observed in several sonographic characteristics between the two groups.

**Table 4:** Comparison of conventional ultrasonographic features.

Parameter	Features	<10 mm (n=47)	≥10 mm (n=56)	^χ2^/t/W	P
Orientation	Horizontal	5(10.63)	30(53.57)	20.996	0.000
	Vertical	42(89.36)	26(46.42)		
Margin	Circumscribed	6(12.76)	28(50)	16.020	0.000
	Ill-defined	41(87.23)	28(50)		
	Extrathyroidal extension	0(0)	0(0)		
Composition	Solid	46(97.87)	35(62.5)	19.035	0.000
	Predominately solid	1(2.12)	21(37.5)		
Echogenicity	Hypoechogenicity	46(97.87)	35(62.5)	19.035	0.000
	Isoechogenicity	1(2.12)	21(37.5)		
Microcalcification	without	15(31.91)	30(53.57)	4.871	0.021
	with	32(68.08)	26(46.42)		
CDFI	0	16(34.04)	7(12.5)	1845.000	0.000
	1	20(42.55)	15(26.78)		
	2	6(12.76)	8(14.28)		
	3	5(10.63)	26(46.42)		
Lymph metastasis	No	40(85.1)	44(78.57)	0.725	0.394
	Yes	7(14.89)	12(21.42)		
Position	Left lobe	16(34.04)	22(39.28)	1.422	0.491
	Right lobe	30(63.82)	34(60.71)		
	Isthmus	1(2.12)	0(0)		
Location	Entire layer	0(0)	9(16.07)	19.536	0.000
	Upper layer	23(48.93)	12(21.42)		
	Middle layer	12(25.53)	28(50)		
	Deep layer	12(25.53)	7(12.5)		

Nodules <10 mm were more frequently characterized by a solid composition (97.87% vs. 62.50%, P < 0.001), ill-defined margins (87.23% vs. 50.00%, P < 0.001), a vertical orientation (89.36% vs. 46.42%, P<0.001), and hypoechoic echogenicity (97.87% vs. 62.50%, P < 0.001). Additionally, microcalcifications were more commonly observed in the <10 mm group (68.08% vs. 46.42%, P = 0.021). CDFI patterns also differed significantly between the groups (P < 0.001). Grade 3 vascularity, indicating rich internal blood flow, was significantly more prevalent in nodules ≥10 mm compared to those <10 mm (46.42% vs. 10.63%), whereas Grade 0 (no detectable blood flow) was more frequent in nodules <10 mm (34.04% vs.12.50%).

In terms of nodule location within the thyroid, a significantly higher proportion of nodules in the ≥10 mm group were located in the middle layer (50.00% vs. 25.53%, P < 0.001), whereas nodules <10 mm were more often located in the upper layer (48.93% vs. 21.42%) (**Table 4**). No significant differences were found between the two groups regarding lymph node metastasis (P = 0.394), lobe position (P = 0.491).

### Comparison of SWE parameters between different nodule size groups

**Figure 2:**
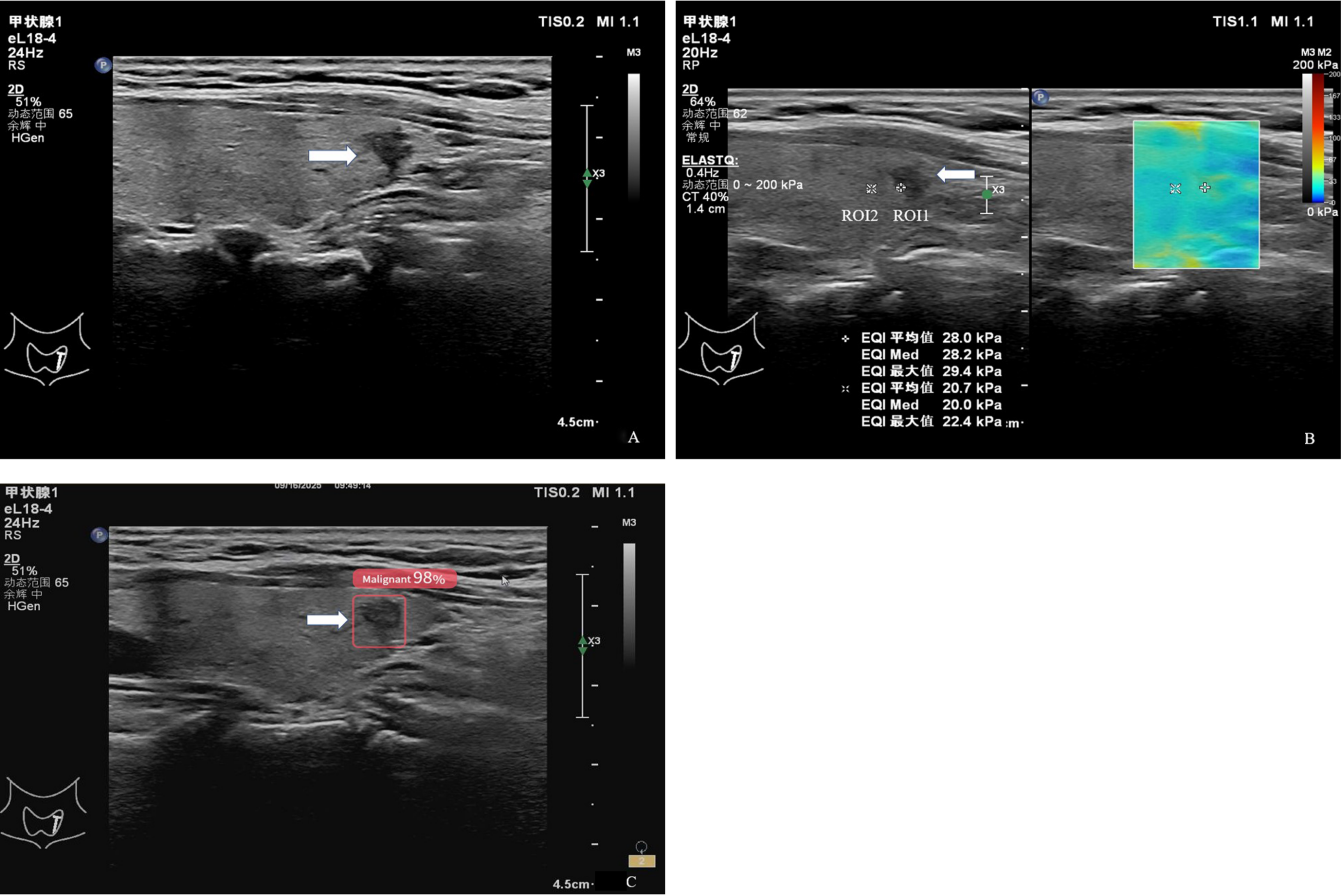
A 56-year-old patient with a thyroid nodule who underwent thyroidectomy. **(A)** Gray-scale ultrasound image shows a purely solid nodule measuring less than 10 mm in maximum diameter, with an irregular shape, non-parallel orientation (aspect ratio > 1), and an unclear margin, without microcalcifications. **(B)** Shear-wave elastography (SWE) image of the same nodule. A 2-mm circular region of interest (ROI 1) was positioned within the stiffest solid portion of the lesion, avoiding calcified or cystic components. A second ROI (ROI 2) was placed in the adjacent normal thyroid parenchyma as a reference. The measured stiffness values for ROI 1 were Emax = 29.4 kPa, Emean = 28.0 kPa, and Emed = 28.2kPa. **(C)** AI-assisted diagnostic system output, indicating the nodule as malignant.

**Figure 3:**
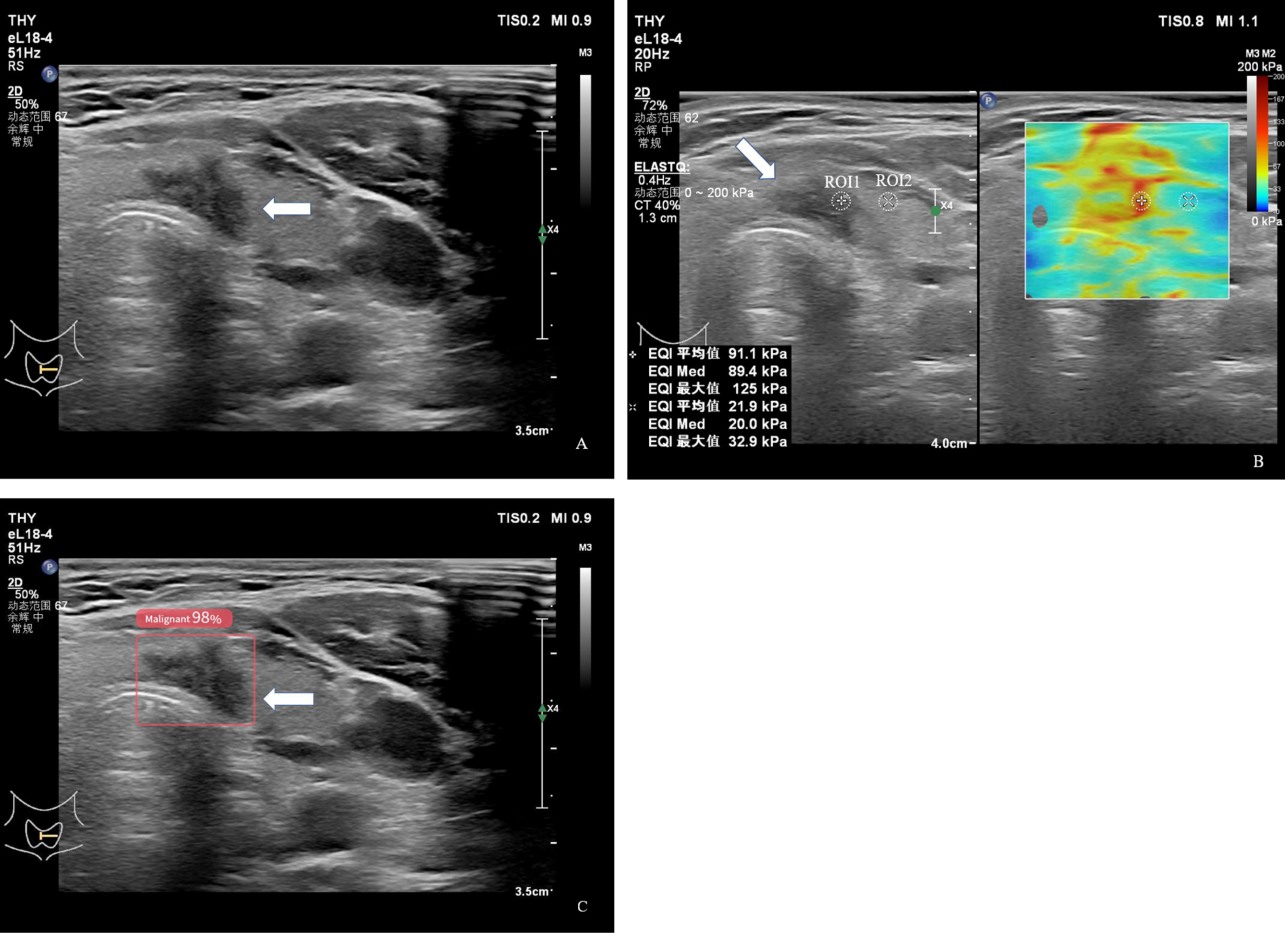
A 45-year-old patient with a thyroid nodule who underwent a thyroidectomy. **(A)** Gray-scale ultrasound image shows a purely solid nodule measuring more than 10 mm in maximum diameter, with an irregular shape, non-parallel orientation (aspect ratio > 1), and an unclear margin, without microcalcifications. **(B)** Shear-wave elastography (SWE) image of the same nodule. A 2-mm circular region of interest (ROI 1) was positioned within the stiffest solid portion of the lesion, avoiding calcified or cystic components. A second ROI (ROI 2) was placed in the adjacent normal thyroid parenchyma as a reference. The measured stiffness values for ROI 1 were Emax = 125 kPa, Emean = 91.1kPa, and Emed = 89.4kPa. **(C)** AI-assisted diagnostic system output, indicating the nodule as malignant.

As shown in **Table 5**, all SWE parameters, including Emax, Emean, and Emed, were significantly higher in the ≥10 mm group than in the <10 mm group. Specifically, the Emax of nodules ≥10 mm was 74.50 ± 44.02 kPa, significantly higher than that of nodules <10 mm (56.44 ± 35.70 kPa, P = 0.026). Similarly, Emean and Emed values in the ≥10 mm group were 53.80 ± 29.79 kPa and 53.62 ± 28.94 kPa, respectively, both significantly greater than the corresponding values in the <10 mm group (41.92 ± 23.15 kPa and 41.53 ± 22.75 kPa; P = 0.028 and P = 0.022, respectively) (**Figures 2**, **3**).

**Table 5:** Comparison of SWE parameters between the two groups.

Parameter	<10mm group	≥10mm group	t	P
Emax	56.44 ± 35.70	74.50 ± 44.02	-2.256	0.026
Emed	41.53 ± 22.75	53.62 ± 28.94	-2.323	0.022
Emean	41.92 ± 23.15	53.80 ± 29.79	-2.227	0.028

### Comparison of diagnosis between AI assisted diagnostic system and C-TIRADS between different nodule size groups

**Table 6** shows the diagnostic distribution of thyroid nodules using the AI system and C-TIRADS classification in the <10 mm and ≥10 mm groups. The AI-assisted system classified 93.61% (44/47) of <10 mm nodules as malignant, significantly higher than the 57.14% (32/56) in the ≥10 mm group (P < 0.001). In contrast, the proportion of nodules classified as benign by AI was notably higher in the ≥10 mm group (41.07%) compared to the <10 mm group (6.38%). Only one nodule in the ≥10 mm group was labeled as “uncertain”.

**Table 6:** Comparison of diagnosis between AI and C-TIRADS of different sizes in the thyroid.

Diagnostic method	Category	<10 mm(n=47)	≥10 mm(n=56)	χ2	P
AI Classification	Malignant	44(93.61)	32(57.14)	18.657	<0.001
	Uncertain	0(0)	1(1.78)		
	Benign	3(6.38)	23(41.07)		
C-TIRADS	1	0(0)	0(0)	19.889	<0.001
	2	0(0)	1(1.78)		
	3	2(4.25)	19(33.92)		
	4A	17(36.17)	9(16.07)		
	4B	18(38.29)	13(23.21)		
	4C	3(6.38)	2(3.57)		
	5	7(14.89)	12(21.42)		
	6	0(0)	0(0)		

For C-TIRADS, no nodules were assigned to category 1 or 6 in either group. Most <10 mm nodules were rated as category 4A (36.17%),4B (38.29%) and 5(14.89%), with few in category 3 (4.25%) or 4C (6.38%). In the ≥10 mm group, however, nodules were more evenly distributed across categories 3 (33.92%), 4A (16.07%), 4B (23.21%) and 5(21.42%), with few in category 4C (3.57%). The difference in the C-TIRADS distribution between the two groups was statistically significant (P < 0.001), with a tendency toward lower C-TIRADS categories observed in larger nodules.

### ROC analysis

Subgroup analyses based on maximum nodule diameter (<10 mm vs ≥10 mm) demonstrated notable differences in diagnostic performance across methods. In the overall cohort, AI showed the highest discriminatory ability (AUC = 0.834), followed by C-TIRADS (AUC = 0.766) and Emax (AUC = 0.747) (**Table 7A**). In nodules <10 mm, most methods exhibited reduced performance, with AI (AUC = 0.654) and C-TIRADS (AUC = 0.524) showing limited diagnostic value. Notably, SWE, particularly Emean, maintained relatively strong discrimination (AUC = 0.821), outperforming Emax (0.659) and Emed (0.652) (**Table 7B**).In nodules ≥10 mm, all methods demonstrated substantial improvement, with AI (AUC = 0.875), C-TIRADS (AUC = 0.834), and Emax (AUC = 0.895) achieving high diagnostic accuracy, the latter representing the best-performing SWE parameter (**Table 7C**).

**Table 7A.** Overall diagnostic performance of different methods.

Diagnostic method	AUC	P	cutoff	Sensitivity	Specificity	Youden Index	PPV	NPV	Accuracy
AI*	0.834	0.000	–	97.10	69.70	0.67	87.01	92.00	88.24
C-TIRADS	0.766	0.000	–	95.71	57.58	0.53	82.72	86.36	83.50
Emax	0.747	0.000	54.90	65.70	78.80	0.45	86.80	51.99	69.90
Emed	0.731	0.000	42.53	62.90	81.80	0.45	88.00	50.97	68.96
Emean	0.743	0.000	37.25	72.90	75.80	0.49	86.47	56.87	73.83
AI+C-TIRADS	0.781	0.000	–	98.57	57.58	0.56	83.13	95.00	85.44
AI+Emax+C-TIRADS	0.781	0.000	–	96.55	70.37	0.67	77.78	95.00	83.93

* One “uncertain” case was excluded from the analysis.

According to the DeLong test, there was no significant difference between AI + C-TIRADS and AI + Emax + C-TIRADS (P = 1.000).

**Table 7B.** Diagnostic performance stratified by nodule size (<10 mm).

Diagnostic method	AUC	P	cutoff	Sensitivity	Specificity	Youden Index	PPV	NPV	Accuracy
AI	0.654	0.226	–	97.56	33.33	0.31	90.91	66.67	89.36
C-TIRADS	0.524	0.848	–	95.12	0.00	-0.05	86.67	0.00	82.98
Emax	0.659	0.214	53.15	46.34	83.33	0.30	95.00	18.52	51.06
Emed	0.652	0.232	44.12	48.80	83.30	0.32	95.23	19.23	53.20
Emean	0.821	0.012	14.60	92.70	33.30	0.26	90.47	40.03	85.12
AI+C-TIRADS	0.512	0.924	–	97.56	0.00	-0.02	86.96	0.00	85.11
AI+Emax+C-TIRADS	0.512	0.924	–	97.56	0.00	-0.02	86.96	0.00	85.11

According to the DeLong test, there was no significant difference between AI + C-TIRADS and AI + Emax + C-TIRADS (P = 1.000).

**Table 7C.** Diagnostic performance stratified by nodule size (≥10 mm).

Diagnostic method	AUC	P	cutoff	Sensitivity	Specificity	Youden Index	PPV	NPV	Accuracy
AI*	0.875	0.000	–	96.43	77.78	0.74	81.82	95.45	87.27
C-TIRADS	0.834	0.000	–	96.55	70.37	0.67	77.78	95.00	83.93
Emax	0.895	0.000	60.58	93.10	77.78	0.71	81.82	91.30	85.71
Emed	0.874	0.000	42.53	96.60	77.80	0.74	82.37	95.52	87.54
Emean	0.885	0.000	28.45	96.60	81.50	0.78	84.87	95.71	89.32
AI+C-TIRADS	0.852	0.000	–	100.00	70.37	0.70	78.38	100.00	85.71
AI+Emax+C-TIRADS	0.852	0.000	–	100.00	70.37	0.70	78.38	100.00	85.71

* One “uncertain” case was excluded from the analysis.

According to the DeLong test, there was no significant difference between AI + C-TIRADS and AI + Emax + C-TIRADS (P = 1.000).

To further evaluate diagnostic optimization, parallel testing was conducted to compare the two combination models (AI plus C-TIRADS and AI plus C-TIRADS plus Emax). Across the overall cohort and both size-based subgroups, the model integrating only AI and C-TIRADS achieved the same AUC as the triple combination (AI plus C-TIRADS plus Emax). DeLong’s test confirmed that no significant difference existed between the two approaches (P = 1.000) (**Tables 7A**–**C**).

## Discussion

The clinical management of thyroid nodules, particularly in the context of varying lesion sizes, remains challenging due to the heterogeneity in diagnostic performance across different imaging modalities (19–22). In this study, we systematically compared the diagnostic efficacy of SWE, C-TIRADS, and AI-assisted diagnostic systems in distinguishing benign from malignant thyroid nodules, stratified by lesion size. Our results demonstrate that the sensitivity, specificity, and the AUC differed significantly both across diagnostic methods and between nodules <10 mm and ≥10 mm. highlighting the importance of adopting size-specific diagnostic strategies in clinical practice.

It should be noted, however, that the high proportion of malignant nodules—particularly among subcentimeter lesions—reflects the tertiary oncology setting of our cohort and may have inflated certain performance metrics such as the positive predictive value. Therefore, our findings should be interpreted as indicative of the relative discriminatory capacity of the evaluated methods, rather than their absolute predictive performance in a general or screening population.

In our study, subcentimeter thyroid nodules had a significantly higher rate of classic malignant ultrasound features, including irregular shape, ill-defined margins, vertical orientation, hypoechogenicity, and microcalcifications, compared to nodules ≥10 mm. These findings are highly consistent with the malignant features emphasized in the latest C-TIRADS guidelines (10) and may partially explain the higher malignancy rate observed in the <10 mm group (87.23% vs. 50.00%). Moreover, previous studies have reported an inverse relationship between thyroid nodule size and malignancy risk, with smaller nodules more frequently exhibiting malignant behavior—consistent with our findings (26). This epidemiological trend underscores the importance of carefully evaluating subcentimeter nodules, particularly those with suspicious imaging characteristics. These results further support the need for size-stratified diagnostic strategies in clinical settings.

Although conventional ultrasound provides essential morphological features of thyroid nodules, such as borders, echogenicity, calcification, and aspect ratio, the C-TIRADS system further classifies these features to assess malignancy risk, they are inherently limited in their ability to reflect the biomechanical properties of thyroid tissue (27). In this context, SWE, an advanced technique capable of quantifying tissue stiffness, offers complementary diagnostic information in the differentiation of benign and malignant thyroid nodules (28). By measuring the propagation speed of shear waves within the tissue to calculate the Young’s modulus, SWE indirectly reflects tissue elasticity, and has demonstrated promising diagnostic performance in the evaluation of various solid tumors (29).

After stratifying nodules by size, our study revealed that the diagnostic performance of SWE varied significantly across different size groups. Previous studies have reported that SWE demonstrates higher diagnostic efficiency in larger nodules and generally recommend Emax as the preferred parameter due to its greater sensitivity to focal stiffness changes (20). Consistent with these findings, our study showed that Emax achieved the highest diagnostic performance in nodules ≥10 mm (AUC = 0.895). Emean also exhibited comparable diagnostic efficacy in this subgroup (AUC = 0.885). In nodules <10 mm, SWE parameters declined overall (Emax 0.659, Emed 0.652), whereas Emean retained relatively strong discriminative performance (AUC = 0.821), highlighting its potential to reflect the overall stiffness pattern of nodules. However, because we did not evaluate measurement repeatability or variability, such as coefficients of variation, we could not further compare the stability or reproducibility of Emean and Emax. Therefore, we report only their diagnostic performance without inferring superiority. Future studies incorporating repeated measurements and larger samples are needed to clarify the reproducibility and clinical applicability of different SWE parameters.

The SWE parameters of the Young’s modulus also differed across nodules of different sizes, which is consistent with previous studies (20). These observed elastic changes may be related to tumor volume–associated pathological alterations such as tissue remodeling, fibrosis, increased cellular density, collagen deposition, local stromal reactions, and reduced vascular compliance (30, 31). These biological changes are particularly common in papillary thyroid carcinoma, which constituted the majority of malignant nodules in our cohort. In contrast, smaller nodules (<10 mm) tended to show lower stiffness parameters. This size-dependent difference may be attributed to a combination of biological and technical factors. Biologically, smaller malignant nodules, especially early-stage papillary carcinomas, may not yet exhibit significant fibrosis or stromal remodeling, resulting in lower tissue stiffness and thus reduced sensitivity of SWE (32). Technically, shear wave propagation may be less stable in small or superficial nodules, reducing the accuracy of stiffness measurements (20).Moreover, subcentimeter nodules are more susceptible to artifacts caused by adjacent anatomical structures or patient motion, which may further compromise image quality (33, 34).

SWE is a non-invasive technique with high operability, providing biomechanical insights into tissue stiffness. However, its diagnostic performance remains inconsistent across studies (35). The 2023 Chinese Guidelines for the Diagnosis and Treatment of Thyroid Nodules and Differentiated Thyroid Cancer (Second Edition) have incorporated elastography, but only weakly recommend it as an adjunct to conventional ultrasound, based on moderate-quality evidence (19). Given these limitations, the application of AI-assisted diagnostic systems in thyroid nodule assessment has been gaining increasing attention.

AI-assisted diagnostic systems have emerged as a promising tool in the evaluation of thyroid nodules, offering high diagnostic accuracy and consistency by leveraging deep learning algorithms trained on large ultrasound datasets (21, 36). In our study, AI demonstrated superior diagnostic performance in nodules ≥10 mm compared to those <10 mm, with AUC values of 0.875 and 0.654, respectively. This suggests that AI performs with greater accuracy in recognizing larger nodules. However, in the ≥10 mm subgroup, Emax achieved a slightly higher AUC than the AI-assisted system. This observation may be explained by the biomechanical properties of larger lesions and the sensitivity of SWE to localized stiffness variations. Moreover, the AI model was applied without additional local retraining, which may have influenced its relative performance. These real-world findings highlight the value of presenting objective results and indicate that further optimization and population-specific adaptation of AI algorithms may still be needed.

To further assess the clinical value of multimodal combinations, this study conducted a parallel analysis of the three parameters with the highest individual AUC performance: AI, C-TIRADS, and Emax. The results indicated that the diagnostic performance of AI + C-TIRADS + Emax was identical to that of AI + C-TIRADS in terms of diagnostic efficacy. This finding suggests a potential diagnostic saturation, where adding additional parameters (such as Emax) does not further enhance diagnostic efficacy. Accordingly, in clinical practice, combining AI with C-TIRADS alone may achieve comparable diagnostic results while avoiding additional resource consumption and equipment dependence. This approach aligns more closely with current healthcare resource allocation and cost-effectiveness considerations, facilitating broader application, especially in resource-limited settings. Notably, the combination of AI and C-TIRADS significantly improved sensitivity both overall and in nodules ≥10 mm, with a slight decrease in specificity; nevertheless, their complementary diagnostic dimensions still suggest a potential synergistic value.

Although the diagnostic performance of AI + C-TIRADS and AI + C-TIRADS + Emax was comparable in the parallel analysis, we further explored why Emax is the preferred parameter in clinical practice. Among the three SWE parameters assessed in this study, Emax demonstrated the best overall AUC performance. Compared to Emean and Emed, Emax more accurately reflects the maximal stiffness of the lesion, and its “peak” feature allows for the establishment of clear diagnostic thresholds, facilitating model integration and standardization. From both a biomechanical and clinical operational standpoint, Emax shows stronger potential for integration. Therefore, even though it did not further improve the performance of the combined model in this study, Emax remains one of the most valuable SWE parameters for future multimodal diagnostic strategies.

## Limitation

This study has several limitations. First, it was conducted at a single center using one ultrasound system, and lacks multicenter or multi-device validation. Second, all participants were recruited from a tertiary oncology hospital, where the malignancy rate was substantially higher than in general populations. This case-mix may introduce selection bias and raise the pretest probability of malignancy, potentially leading to overestimation of prevalence-dependent metrics such as PPV and AUC. Third, the AI-assisted diagnostic system assessed in this study is specific to a single platform, and its performance may vary across different devices or software. Further multicenter studies involving diverse patient populations and equipment are needed to strengthen the generalizability of our findings.

## Conclusion

In conclusion, nodule size significantly affects the diagnostic performance of AI, C-TIRADS, and SWE. In nodules ≥10 mm, both AI and C-TIRADS showed strong performance, with Emax being the most effective SWE parameter. The combination of AI and C-TIRADS yielded comparable efficacy to AI + C-TIRADS + Emax, suggesting limited added value of SWE in larger nodules. For subcentimeter nodules, diagnostic performance declined across all methods, including combined approaches; however, Emean retained some discriminative ability, indicating its potential to reflect overall stiffness patterns in small nodules. These results highlight the importance of selecting appropriate imaging parameters and implementing size-stratified diagnostic strategies.

## Data Availability

The raw data supporting the conclusions of this article will be made available by the authors, without undue reservation.
